# Contact Endoscopy as a Novel Technique in the Detection and Diagnosis of Mucosal Lesions in the Head and Neck: A Brief Review

**DOI:** 10.1155/2011/196302

**Published:** 2010-12-16

**Authors:** Christopher Szeto, Bret Wehrli, Fiona Whelan, Jason Franklin, Anthony Nichols, John Yoo, Kevin Fung

**Affiliations:** ^1^Division of Head and Neck Oncology and Reconstructive Surgery, Department of Otolaryngology - Head and Neck Surgery, Victoria Hospital, University of Western Ontario, London, Ontario, Canada N6A 5W9; ^2^Department of Pathology, University Hospital, University of Western Ontario, London, Ontario, Canada N6A 5C1; ^3^Division of Head and Neck Oncology, Department of Otolaryngology - Head and Neck Surgery, Fremantle Hospital & Health Service, WA 6959, Australia

## Abstract

*Background*. There are a variety of described noninvasive optical detection techniques for evaluation of head and neck mucosal lesions. Contact endoscopy is a promising method of *in vivo* microscopic examination whereby a rigid telescope is placed on a previously dye-stained mucosa allowing evaluation of the superficial cell layers of the epithelium. This technique produces real-time, magnified images of cellular architecture of surface mucosa comparable to histology without the need for biopsy. In this review, we will briefly summarize the efficacy of CE in the detection of precancerous and cancerous mucosal lesions and its potential as a novel technique in early diagnosis, monitoring, and preoperative assessment of mucosal lesions of the head and neck. *Methods*. PUBMED, MEDLINE, and COCHRANE search revealed five prospective articles on contact endoscopy for the diagnosis of mucosal lesions in the head and neck. *Results*. The literature search yielded five prospective studies examining contact endoscopy for the diagnosis of benign versus malignant head and neck mucosal lesions. These reported a sensitivity and specificity of 77–100%, specificity of 66–100% and an accuracy of 72–92%. *Conclusion*. Contact endoscopy is a promising optical technology that may be a useful adjunct in the evaluation and diagnosis of benign and malignant head and neck mucosal lesions. Future prospective randomized double-blind studies of this detection method are required.

## 1. Introduction

The vast majority of cancers in the oral cavity and in the head and neck are squamous cell carcinomas (SCCs). It is the sixth most common cancer worldwide, and its incidence is rising in industrialized nations [1, 2]. Head and neck cancer is a major cause of morbidity and mortality. Many cancers of the head and neck arise from precancerous lesions such as leukoplakia. Some studies quote leukoplakia as having a 10% chance of transformation into carcinoma. Similarly, other benign lesions of the oral cavity such as lichen planus may have a prevalence of 0.5–2% in the general population and may have a risk of malignant transformation of 1% [3]. Thus, early detection and diagnosis of suspicious mucosal lesions is essential. 

Many benign oral mucosal lesions are not cancerous which presents a clinical dilemma to the physician. Furthermore, precancerous lesions such as leukoplakia may exhibit mild structural alterations in the mucosa that can be difficult to distinguish from normal healthy tissue. Currently, obtaining histopathology via biopsy is the gold standard of diagnosis; however, this procedure can pose significant morbidity to the patient such as the risk of bleeding, wound infection, and potentially impairment of speech and swallowing if multiple biopsies are performed.

Moreover, it becomes a clinical challenge to monitor patients for progression of diffuse dysplasia or leukoplakia, and many of them may require multiple biopsies over many years. The discomfort of biopsy and compromisation of tissue integrity can lead to problems with future biopsy interpretation or in the case of laryngeal biopsy, considerable problems in individuals with high vocal demands [4]. Subsequently, any technique that can yield histopathological information without injuring tissue has obvious advantages over biopsy.

Detailed examinations of the texture, color, contour, and extent of mucosal lesions have been performed utilizing many instruments such as the Hopkins' rod-lens scopes, flexible endoscopes, direct laryngoscopes, and advances in microlaryngoscopic visualization techniques. However, these methods are limited by their inability to provide histopathological data during the clinical examination.

As a result, over the last decade, technological advances in optical imaging detection techniques have emerged with a variety of methods employed to facilitate detailed examination and provision of histopathological information of mucosal lesions. Examples of such novel optical techniques include: aminolevulinic acid-induced fluorecence, autofluorescence, confocal endomicroscopy, and contact endoscopy.

Aminolevulinic acid-induced fluorescence is a technique whereby neoplastic cells undergo preferential fluorescence after aminolevulinic acid (ALA) has been applied to the mucosa surface. In the presence of ALA, tumors have selective accumulation of protoporphyrin which can be differentiated from healthy tissue. Once this “dye-like” substance has been applied, mucosa containing neoplastic cells will fluoresce orange red and normal mucosa will retain the normal green fluorescence. Coupled with autofluorescence, several authors have noted that these techniques can diagnose laryngeal carcinoma and dysplasia with good accuracy [5].

Autofluorescence was first described in identification of neoplastic cells of the larynx by Harris et al. [6]. Tissue fluorescence is induced by short-wave blue light of the visible spectrum. Certain molecules then transform into photonic energy, which is emitted as long-wave scattered light which can be detected. Each molecule has a characteristic fluorescence spectrum dependent on the excitation light. These fluorescent molecules are called fluorophores. The autofluorescence imaging method detects the fluorecence given off by the different concentrations of fluorophores seen in normal and neoplastic mucosa. Normal healthy mucosa fluoresces bright green while neoplastic mucosa appears red violet [7]. Thus, autofluorescence videoendoscopy for photodiagnosis of head and neck squamous cell carcinomas has been described as being quite accurate with good sensitivity and specificity in several studies [6–16].

Unlike ALA and autofluorescence where histological detail is not appreciated, other optical techniques such as narrow-band imaging endoscopy (NBIE) allows increased visualization of histological detail. NBIE uses filtered light with wavelengths preferentially corresponding to peaks of absorption of hemoglobin to enhance superficial neoplasms based on their neoangiogenic pattern. These light wavelengths penetrate superficial mucosal and deep submucosal layers to enhance capillary and submucosal vasculature. The obtained image is further enhanced by using high-definition television (HDTV) [17]. Carcinomas can then be identified based on the changes in the microvascular pattern of the mucosal lesion. Several studies have shown good sensitivity, specificity, negative and positive predictive value, and accuracy in detection of squamous cell carcinoma of the oral cavity, oropharynx, larynx, and esophagus [17–20].

However, instead of solely relying on neoangiogenic patterns for diagnosis of carcinoma, further histological detail can be obtained with the use of confocal endoscopy which is an *in vivo* optical imaging method whereby mucosal lesions can undergo significant magnification to allow examination of cellular histology. This technique also allows reconstruction of three-dimensional structures based on the acquired images. Utilization of various stains to help highlight cellular structures has been tried by some authors to distinguish normal from invasive carcinoma cells. The utility of this new technology is highlighted in its capability to distinguish between benign or low-grade mucosal dysplasia thereby potentially reducing unnecessary biopsies [21]. 

Contact endoscopy is another novel noninvasive optical diagnostic imaging method that allows *in vivo* and in situ examination of the cellular architecture of the superficial layers of the mucosal epithelium. Magnified images are obtained using Hopkins' rod-lens endoscope placed on the surface of the dye stained mucosal tissue. This technique allows assessment of precancerous and cancerous lesions *in vivo* and has significant potential in the histopathologic diagnosis of many suspicious head and neck mucosal lesions without tissue biopsy. 

CE was originally described and used by Hamou in 1979 as a technique for visualization of cervical and uterine epithelial cells for screening and diagnosis of cervical and uterine pathology [22]. The first reported use of CE in otolaryngology head and neck surgery was by Andrea et al. as a diagnostic tool in the evaluation of various pathologies in the larynx in the 1990s [4, 23–30]. They were able to visualize and diagnose laryngeal mucosal pathology from the magnification of vocal fold epithelium and microvasculature during microlaryngoscopy after staining the vocal cords with methylene blue dye. 

**Figure 1:**
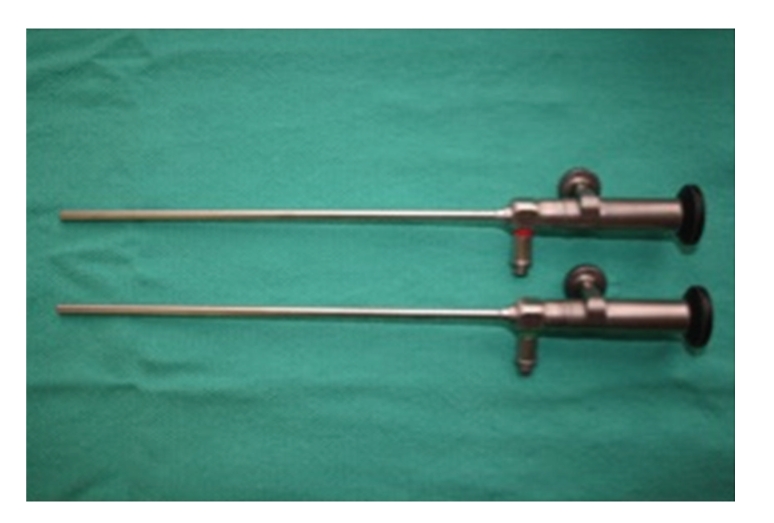
Top (zero-degree) and bottom (thirty-degree) contact endoscopes.

**Figure 2:**
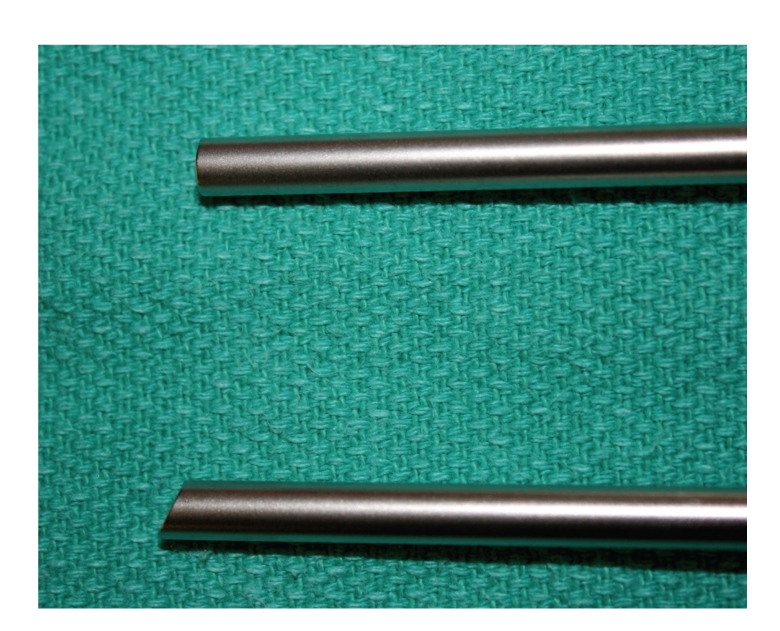
Closeup of endoscope tips. Top (zero-degree) and bottom (thirty-degree) contact endoscopes.

Current contact microlaryngoscopes come in a variety of lengths, diameters and viewing angles. Diameters, of these scopes come as either 4 mm or 5.5 mm and lengths of 23 cm and 18 cm. Straight forward (O°) and Forward-Oblique telescopes (30°) are also available, and all are capable of 1x, 60x, and 150x magnification. These endoscopes require a high intensity xenon light source, and images can be digitally captured for real-time photographic and video documentation, Figures 1 and 2.

**Figure 3:**
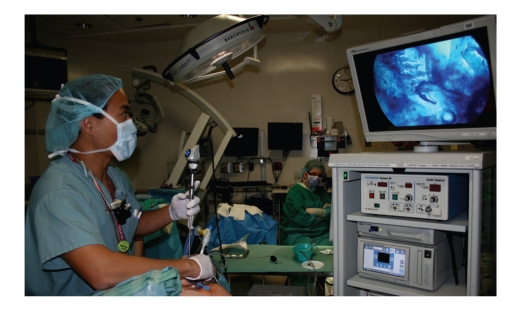
An otolaryngologist performing contact endoscopy of an oral mucosal lesion.

**Figure 4:**
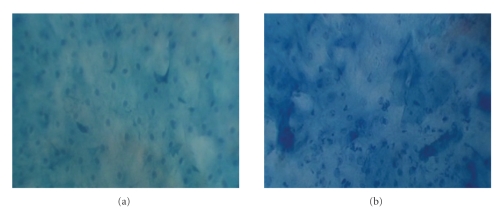
Images (150x magnification) of a benign (normal mucosa on pathology) and malignant (squamous cell carcinoma on pathology) oral cavity lesion demonstrating magnified cellular architecture as acquired by contact endoscopy.

The most basic technique of CE involves staining of the superficial cells of the mucosa with a contrast dye, 1% methylene blue (MB) after which the magnifying endoscope (Karl Storz 8715 AA, Tuttlingen, Germany) 0° is then placed in contact against the mucosal surface, and the documented magnified cytological images (at 60x or 150x) are then recorded, Figure 3. Both a cytopathologist and an otolaryngologist can then assess these images, comparable to histology, Figure 4. Contact endoscopy and its efficacy in head and neck oncology, advantages, limitations, and future potential diagnostic utility will be briefly reviewed in this article.

## 2. Methods

The literature search was conducted using the following key terms: “contact endoscopy”, “contact microlaryngoscopy”, “Aminolevulinic acid induced fluorecence”, “autofluorescence”, “confocal endomicroscopy”, “oral mucosa”, “oral cavity”, ”larynx”, “oropharynx”, “hypopharynx”, “head and neck carcinoma”, “leukoplakia”, and “lichen planus.” Significant publications were identified using MEDLINE, COCHRANE and PUBMED databases. Relevant search terms and combinations using Boolean operators were performed, and relevant article selection was limited to the prospective, human and English studies without restriction to year of publication. All appropriate article references were searched and cross-referenced.

## 3. Results

Five prospective articles were examined. Efficacy data from these studies are summarized in Table 1.

Warnecke et al. [30] examined 42 consecutive patients at a tertiary care center with suspicious lesions of larynx, pharynx, and esophagus under general anesthesia. Indication for endoscopy was a tentative clinical diagnosis of malignant tumor of oropharynx. All were biopsied postendoscopy. The results obtained by the cytopathologist and otolaryngologist were based on images generated from the CE. The histopathology obtained was considered the gold standard. All of the samples obtained were blinded. They found that the more experienced the examiner, the higher the sensitivity of CE was in the diagnostic differentiation of benign versus malignant mucosal lesions.

Cikojević et al. [4] examined the utility of CE in intraoperative diagnosis of laryngeal pathology. They included 142 patients undergoing microlaryngoscopy at their institution with various laryngeal diseases all underwent CE and subsequent biopsy for histopathological diagnosis. All malignant lesions identified by CE was confirmed by histopathology, but CE did not identify malignancy in 10 patients diagnosed histopathologically thus giving CE a sensitivity of 79.6%, specificity of 100%, and accuracy of 93%.

Tarnawski et al. [31] examined 54 patients with various laryngeal pathology intraoperatively during microlaryngoscopy. CE was performed, and biopsies were taken from all patients for histopathological diagnosis. Their results were based on computer-assisted analysis of all CE images based certain nuclear morphometric parameters to determine benign from malignant lesions. Thus, based on their computer-assisted analysis of CE images, their sensitivity was 91% and specificity 81%.

Pak et al. [32] prospectively examined 64 patients with previous irradiation for nasopharyngeal carcinoma (NPC). All patients were examined with contact rhinoscopes under local anesthesia and biopsy of the area under examination was done. In all 5 cases of malignancy, CE and histological diagnosis directly correlated with each other.

Most significantly, for the prediction of persistent and recurrent disease, sensitivity and specificity for CE was 100% with an accuracy of 92.1%. 

Finally, Arens et al. [26] pilot study examined 83 patients using both autofluorescence and contact endoscopy during microlaryngoscopy. For contact endoscopy, the calculated sensitivity was 94.7, specificity of 95.5 and an accuracy of 94%.

In summary, authors of the above prospective trials have obtained the following results: a sensitivity of 79–100%, a specificity of 81–100%, and an accuracy of 88–94%. Overall, it appears that sensitivity, specificity, and accuracy of CE are similar across the trials.

## 4. Discussion

Since the development of contact endoscopy, this technology has been used successfully by several authors in analyzing and diagnosing various pathologies of the larynx, oral cavity, oropharynx, and nasopharynx via “real-time” examination of mucosal cytological detail [4, 26–31, 33–36]. Despite its introduction into otolaryngology, CE has yet to find a place in routine clinical practice despite its potential advantages.

From the above clinical trials, CE appears to have good sensitivity, specificity, and accuracy as a noninvasive method for distinguishing between benign and malignant mucosal lesions in the head and neck. However, some authors state that it may be difficult for CE to detect mild (grade I) mucosal dysplasia because most of the cellular anomalies occur in at the level of the basal epithelium, and this technique can only examine cellular architecture found at the superficial epithelial layers [4, 21]. 

Despite this limitation, other authors have found CE diagnosis to correlate well with histopathological findings. Most significantly, CE accurate ability to diagnose and tease out the histological differences between squamous metaplasia, atypia, and carcinoma even in the presence of irradiated mucosa was highlighted by the study performed by Pak et al. [32]. At present, most authors seem to agree that it has significant potential as a noninvasive detection method that could play a role as a future substitute for histological examination.

There are several advantages of contact endoscopy. Most significantly, it offers a noninvasive, rapid, and repeatable *in vivo* assessment of the cytological architecture while avoiding the need for an invasive biopsy and its associated risks. CE provides immediate results, with the possibility of examining multiple mucosal areas in a short time. CE can also assess a wider surface mucosal area, providing more information than a selected histological section taken by biopsy [30]. It also avoids tissue damage and alteration of cellular architecture which may occur in the biopsy and histological preparation [4]. This noninvasive technique also helps to direct the site of biopsy by identifying areas with cellular atypia and thus avoiding the need for multiple biopsies. Subsequently, this results in a dramatic improvement of the diagnostic yield of the biopsy [32]. Other potential roles of CE include the rapid diagnosis of benign and malignant mucosal lesions in an outpatient or operating room setting, surveillance, guided biopsies, and intraoperative evaluation of tumor resection margins. 

Despite its advantages, CE does have its limitations. Most notably, CE can only evaluate the most superficial cell layer of the mucosal epithelium. This is most likely due to a number of factors including (i) poor penetration of methylene blue which only stains a few superficial layers, (ii) short focal distance of the scope (i.e., CE can only assess to a depth of 80 um at 60x magnification and 30 um at 150x magnification), and (iii) optical artifact at high magnification due to glare from light reflected from cells not in focus. Subsequently, assessment of submucosal lesions or lesions occupying deeper cell layers becomes more difficult [4, 27, 28, 30, 32, 33].

The lack of depth of penetration prevents the evaluation of important histological information especially when vertical extent of dysplasia is crucial in distinguishing the different grades of dysplasia from carcinoma in situ and invasive carcinoma. As a result, these factors could affect the sensitivity of CE, thus accounting for some of the false negative diagnostic results noted by authors. The potential impact of CE missing a malignant lesion needs to be taken into consideration if this technology is to one day substitute histopathology. Future investigation into better penetrating dyes, advances in digital optics, and image enhancements will eventually allow better vertical staining and increased resolution of the deeper cell layers which would translate CE in becoming a much more sensitive and accurate diagnostic tool [31]. 

A pilot study conducted at our institution also investigated some of the limitations and potential advantages of CE in the evaluation of head and neck mucosal lesions. From our preliminary experience, technical difficulties with line of sight, access difficulties to mucosal surfaces, scope positioning, and problems with consistent image quality due to artifact were consistent with those found by previous authors [30, 33]. 

Our pilot study also demonstrated that although CE is a simple, rapid, repeatable, noninvasive examination performed with standard equipment, there is a learning curve associated with its use. However, once one is accustomed with this detection system, CE can be performed almost as quickly as an outpatient flexible fiberoptic nasopharyngoscopic examination. 

In conclusion, *in vivo* assessment of head and neck mucosal pathology may be applied to (i) early detection of premalignant and malignant lesions, (ii) serial follow-up examinations of suspicious lesions such as leukoplakia and lichen planus, and (iii) assessment of resection margins. Despite its limitations, CE represents a promising optical technology that may afford reliable, accurate, and noninvasive *in vivo* assessment of cytological pathology. Prospective investigation with CE is currently ongoing at our institution and necessitates close collaboration between otolaryngologists and pathologists. We hypothesize that future study will demonstrate improved sensitivity, specificity, and accuracy of contact endoscopy in the diagnosis of head and neck tumors.

## Figures and Tables

**Table 1:** Summary of efficacy data from prospective contact endoscopy trials.

Author	Study type	Number of patients	Number of males (M) and females (F)	Average age (age range)	Type of institution	Head and neck subsites	Type of lesions examined	Sensitivity %	Specificity %	Accuracy %
Warnecke et al. [30]	Prospective	42	M = 30 F = 12	55.6 (21–76)	Tertiary	Pharynx, hypopharynx, larynx	Normal and inflamed mucosa, dysplasia, SCC	90	93.8	88
Cikojević et al. [4]	Prospective	142	M = 101 F = 41	N/A (19–81)	Tertiary	Larynx	Benign, hyperplasia, dysplasia (grades I, II, III), papilloma, CIS, SCC	79.6	100	93
Tarnawski et al. [31]	Prospective	54	M = 22 F = 17	51.9 (47–69)	Tertiary	Larynx	Normal mucosa, mild & severe dysplasia, SCC	91	81	N/A
Pak et al. [32]	Prospective	64	M = 54 F = 10	42 (21–77)	Tertiary	Nasopharynx	Metaplasi, atypia, granulation tissue, carcinoma	100	100	92.1
Arens et al. [26]	Prospective	83	N/A	N/A	Tertriary	Larynx	Normal mucosa, dysplasia (grades I, II, III).	94.7	95.5	94

N/A=not available; CIS=carcinoma in situ; SCC=squamous cell carcinoma.

## References

[1] Sankaranarayanan R, Masuyer E, Swaminathan R, Ferlay J, Whelan S (1998). Head and neck cancer: a global perspective on epidemiology and prognosis. *Anticancer Research*.

[2] Duvvuri U, Myers JN (2009). Cancer of the head and neck is the sixth most common cancer worldwide. *Current Problems in Surgery*.

[3] Upile T, Jerjes W, Kafas P, Angouridakis N, Hopper C (2008). The novel use of the micro-endoscope to diagnose oral lichen planus: a case study. *Surgery Journal*.

[4] Cikojević D, Glunčić I, Pešutić-Pisac V (2008). Comparison of contact endoscopy and frozen section histopathology in the intra-operative diagnosis of laryngeal pathology. *Journal of Laryngology and Otology*.

[5] Csanády M, Kiss JG, Iván L, Jóri J, Czigner J (2004). ALA (5-aminolevulinic acid)-induced protoporphyrin IX fluorescence in the endoscopic diagnostic and control of pharyngo-laryngeal cancer. *European Archives of Oto-Rhino-Laryngology*.

[6] Harries ML, Lam S, MacAulay C, Qu J, Palcic B (1995). Diagnostic imaging of the larynx: autofluorescence of laryngeal tumours using the helium-cadmium laser. *Journal of Laryngology and Otology*.

[7] Arens C, Dreyer T, Glanz H, Malzahn K (2004). Indirect autofluorescence laryngoscopy in the diagnosis of laryngeal cancer and its precursor lesions. *European Archives of Oto-Rhino-Laryngology*.

[8] Arens C, Reußner D, Woenkhaus J, Leunig A, Betz CS, Glanz H (2007). Indirect fluorescence laryngoscopy in the diagnosis of precancerous and cancerous laryngeal lesions. *European Archives of Oto-Rhino-Laryngology*.

[9] Malzahn K, Dreyer T, Glanz H, Arens C (2002). Autofluorescence endoscopy in the diagnosis of early laryngeal cancer and its precursor lesions. *Laryngoscope*.

[10] Rydell R, Eker C, Andersson-Engels S, Krogdahl A, Wahlberg P, Svanberg K (2008). Fluorescence investigations to classify malignant laryngeal lesions in vivo. *Head and Neck*.

[11] Delank W, Khanavkar B, Nakhosteen JA, Stoll W (2000). A pilot study of autofluorescent endoscopy for the in vivo detection of laryngeal cancer. *Laryngoscope*.

[12] Baletic N, Petrovic Z, Pendjer I, Malicevic H (2004). Autofluorescent diagnostics in laryngeal pathology. *European Archives of Oto-Rhino-Laryngology*.

[13] Mostafa BE, Shafik AG, Fawaz S (2007). The role of flexible autofluorescence laryngoscopy in the diagnosis of malignant lesions of the larynx. *Acta Oto-Laryngologica*.

[14] Žargi M, Fajdiga I, Šmid L (2000). Autofluorescence imaging in the diagnosis of laryngeal cancer. *European Archives of Oto-Rhino-Laryngology*.

[15] Arens C, Reußner D, Neubacher H, Woenckhaus J, Glanz H (2006). Spectrometric measurement in laryngeal cancer. *European Archives of Oto-Rhino-Laryngology*.

[16] Paczona R, Temam S, Janot F, Marandas P, Luboinski B (2003). Autofluorescence videoendoscopy for photodiagnosis of head and neck squamous cell carcinoma. *European Archives of Oto-Rhino-Laryngology*.

[17] Piazza C, Cocco D, Del Bon F (2010). Narrow band imaging and high definition television in evaluation of oral and oropharyngeal squamous cell cancer: a prospective study. *Oral Oncology*.

[18] Piazza C, Cocco D, De Benedetto L, Bon FD, Nicolai P, Peretti G (2010). Role of narrow-band imaging and high-definition television in the surveillance of head and neck squamous cell cancer after chemo- and/or radiotherapy. *European Archives of Oto-Rhino-Laryngology*.

[19] Piazza C, Cocco D, De Benedetto L, Del Bon F, Nicolai P, Peretti G (2010). Narrow band imaging and high definition television in the assessment of laryngeal cancer: a prospective study on 279 patients. *European Archives of Oto-Rhino-Laryngology*.

[20] Watanabe A, Taniguchi M, Tsujie H, Hosokawa M, Fujita M, Sasaki S (2008). The value of narrow band imaging endoscope for early head and neck cancers. *Otolaryngology. Head and Neck Surgery*.

[21] Hughes OR, Stone N, Kraft M, Arens C, Birchall MA (2010). Optical and molecular techniques to identify tumor margins within the larynx. *Head & Neck*.

[22] Hamou J, Salat-Baroux J, Coupez F, De Brux J (1984). Microhysteroscopy: a new approach to the diagnosis of cervical intraepithelial neoplasia. *Obstetrics and Gynecology*.

[23] Andrea M, Dias O, Macor C, Santos A, Varandas J (1997). Contact endoscopy of the nasal mucosa. *Acta Oto-Laryngologica*.

[24] Andrea M, Dias O, Santos A (1995). Contact endoscopy of the vocal cord: normal and pathological patterns. *Acta Oto-Laryngologica*.

[25] Andrea M, Dias O, Santos A (1995). Contact endoscopy during microlaryngeal surgery: a new technique for endoscopic examination of the larynx. *Annals of Otology, Rhinology and Laryngology*.

[26] Arens C, Glanz H, Dreyer T, Malzahn K (2003). Compact endoscopy of the larynx. *Annals of Otology, Rhinology and Laryngology*.

[27] Carriero E, Galli J, Fadda G, Di Girolamo S, Ottaviani F, Paludetti G (2000). Preliminary experiences with contact endoscopy of the larynx. *European Archives of Oto-Rhino-Laryngology*.

[28] Wardrop PJC, Sim S, McLaren K (2000). Contact endoscopy of the larynx: a quantitative study. *Journal of Laryngology and Otology*.

[29] Dedivitis RA, Pfuetzenreiter EG, Guimaraes AV (2009). Contact endoscopy of the larynx as an auxiliary method to the surgical margins in frontolateral laryngectomy. *Acta Otorhinolaryngologica Italica*.

[30] Warnecke A, Averbeck T, Leinung M (2010). Contact endoscopy for the evaluation of the pharyngeal and laryngeal mucosa. *Laryngoscope*.

[31] Tarnawski W, Fraczek M, Jeleń M, Krecicki T, Zalesska-Krecicka M (2008). The role of computer-assisted analysis in the evaluation of nuclear characteristics for the diagnosis of precancerous and cancerous lesions by contact laryngoscopy. *Advances in medical sciences*.

[32] Pak MW, To KAF, Leung SF, van Hasselt CA (2002). In vivo diagnosis of persistent and recurrent nasopharyngeal carcinoma by contact endoscopy. *Laryngoscope*.

[33] Pelucchi S, Bianchini C, Travagli M, Pastore A (2007). Contact endoscopy of the oral mucosa: preliminary results. *Acta otorhinolaryngologica Italica*.

[34] Sone M, Sato E, Hayashi H, Fujimoto Y, Nakashima T (2006). Vascular evaluation in laryngeal diseases: comparison between contact endoscopy and laser doppler flowmetry. *Archives of Otolaryngology. Head and Neck Surgery*.

[35] Pak MW, To KAF, Leung SF, van Hasselt CA (2001). In vivo diagnosis of nasopharyngeal carcinoma using contact rhinoscopy. *Laryngoscope*.

[36] Xiaoming H, Haiqiang M, Manquan D (2001). Examination of nasopharyngeal epithelium with contact endoscopy. *Acta Oto-Laryngologica*.

